# Intracellular Vesicle Transport Impairment as a Candidate Systems-Level Bottleneck in Chronic Diabetic Foot Ulcers: Network Medicine Identifies KIF13A as a Potential Therapeutic Vulnerability

**DOI:** 10.3390/biomedicines14051140

**Published:** 2026-05-18

**Authors:** Haitao Ren, Yongan Xu

**Affiliations:** 1 Department of Vascular Surgery, The Second Affiliated Hospital of Zhejiang University School of Medicine, Hangzhou 310009, China; 2 Department of Emergency, The Second Affiliated Hospital of Zhejiang University School of Medicine, Hangzhou 310009, China

**Keywords:** diabetic foot ulcer, wound healing, vesicle transport, KIF13A, network medicine, virtual gene knockout, recycling endosome, drug repurposing, epalrestat, functional decoupling

## Abstract

**Background**: Growth factor therapy often fails in diabetic foot ulcers (DFUs). The reason remains unclear. Standard differential expression analysis may miss functionally critical genes with modest expression changes. **Methods**: We performed a secondary computational analysis of a longitudinal DFU transcriptomic dataset (Dryad; 17 patients, 117 serial biopsy samples, 12-week follow-up). Co-expression networks were built separately for healed (*n* = 37) and non-healed (*n* = 80) samples. Virtual gene knockout (VGK) was used to rank genes by topological impact on network cohesion. Single-cell analysis (GSE165816) assessed the association between endogenous KIF13A expression and keratinocyte migration-related signatures. A conceptual Hill-equation simulation was used to illustrate the transport-signaling threshold relationship. Drug repurposing used DSigDB enrichment. An independent bulk DFU cohort (GSE134431) was used for external validation. **Results**: KIF13A showed no differential expression (log_2_FC = 0.173, *p* = 0.263) yet ranked first by VGK topological impact. In keratinocytes, high KIF13A expression correlated with greater migration scores versus zero-detection cells (*p* = 0.0058). A clear threshold effect emerged: below the 30th expression percentile, EGF, PDGF, and FGF pathway activation scores remained near baseline. In a structural-equation model, transport activity negatively predicted inflammation (standardized β = −0.92, *p* < 0.001). HIF1A showed the strongest positive correlation with KIF13A in keratinocytes (Spearman ρ = 0.26, *p* < 0.001), and FOS showed a negative correlation in the single-cell analysis (ρ = −0.16, *p* < 0.001) and in the bulk longitudinal cohort (ρ = −0.32, *p* < 0.001, *n* = 117). Recurrent AKR1B1-related drug signatures nominated the aldose-reductase pathway, and epalrestat was therefore prioritized as a hypothesis-generating candidate compound rather than a direct top-ranked enrichment hit. External validation confirmed consistent upregulation of KIF13A (Fold-Change = 1.58, adj. *p* = 0.0075), EPN1, and CLIP1 in DFU tissue. Despite population-level upregulation, a subset of cells fell below the functional signaling threshold. **Conclusions**: These computational findings suggest that KIF13A-associated vesicle transport impairment may represent a candidate systems-level bottleneck for growth-factor responsiveness in DFUs, a network-level pattern not captured by standard differential-expression analysis. Epalrestat, an AKR1B1 inhibitor prioritized through recurrent AKR1B1-related drug signatures, is presented as a candidate compound for further evaluation. As the present analysis is observational and computational, the findings should be interpreted as hypothesis-generating; experimental perturbation studies and prospective clinical validation are required.

## 1. Introduction

Diabetic foot ulcers (DFUs) are leading causes of amputation [1], with mortality rates often exceeding those of many cancers [2,3,4]. Despite new treatments, we still lack effective ways to close these wounds. Growth factor-based therapies have shown potential to accelerate DFU healing in selected settings [5,6,7]. Though growth factor deficiency is a known factor [8], many patients fail to heal even with aggressive supplementation. This suggests the problem is not just a lack of external signals, but a failure in how cells process them.

Healing requires cells to move and rebuild the tissue matrix, which depends on transporting receptors and adhesion molecules to the cell surface [9]. Standard analysis often misses regulators that do not show massive expression changes. Network medicine treats the transcriptome as an interconnected system, not a collection of independent genes [10,11,12]. In this topology-driven framework, a gene’s functional weight is determined less by its expression level than by its network position. Virtual gene knockout (VGK) analysis builds on this logic by simulating node removal [13]. Regulators whose loss disproportionately destabilizes the system can thereby be identified—even when their transcript abundance appears unremarkable. By using VGK, we can identify these hidden drivers by seeing how their absence disrupts cellular networks. Tools such as scTenifoldKnk provide a computational framework for prioritizing candidate gene-perturbation effects without the immediate cost of in vivo experiments [13,14].

Beyond impaired growth-factor signaling, chronic DFU wounds are characterized by persistent inflammation and altered immune-cell composition. Macrophage polarization, dendritic-cell activity, and regulatory immune populations shape keratinocyte migration, extracellular-matrix remodeling, and re-epithelialization. Whether intracellular transport activity in epithelial cells is coupled to this inflammatory milieu is unknown and is one of the questions the present analysis addresses.

This study integrates network topology analysis, single-cell virtual perturbation, data-driven signal transduction modeling, and network pharmacology to analyze longitudinal transcriptomic data from DFU patients and to identify candidate cellular defects associated with impaired growth-factor responsiveness.

## 2. Materials and Methods

### 2.1. Study Design and Data Sources

This study is a secondary computational analysis of multiple publicly available transcriptomic resources. The primary discovery cohort is a longitudinal bulk RNA-seq dataset of DFU wound-edge biopsies obtained from the Dryad Digital Repository (doi:10.5061/dryad.2v6wwpzzc; raw reads at SRA PRJNA1200081), which is used for co-expression network construction, virtual gene knockout, transport-module and inflammation scoring, and structural-equation modeling. The single-cell RNA-seq dataset GSE165816 is used for keratinocyte clustering, KIF13A expression stratification, and migration scoring. The bulk RNA-seq dataset GSE134431 is used as the independent external validation cohort. The DSigDB and LINCS L1000 databases are used for drug-signature enrichment. The Dryad cohort is described below; the use of each additional resource is described in Section 2. The DFU patients underwent a 12-week follow-up. Healing was defined as complete wound closure within 12 weeks. Data are presented as n, mean (SD), or median (range), as appropriate. The cohort was obtained from the Dryad Digital Repository (doi:10.5061/dryad.2v6wwpzzc). Wound-edge tissue was collected during serial debridement at the Northern California VA Medical Center (2021–2022). Healing was defined as complete wound closure within 12 weeks. Detailed patient demographics (age, sex, diabetes duration, and HbA1c) were not available in the public dataset; the original study collected samples under an exempt VA IRB protocol for discarded tissue. *p*-values for biopsy timing were calculated using the Wilcoxon rank-sum test. RNA-seq data were processed using the CogentAP pipeline (Takara Bio v2.0) with alignment to GRCh38 via STAR and gene counting via featureCounts.

### 2.2. Co-Expression Network Construction and Virtual Gene Knockout

This study employed network analysis to identify key genes influencing wound healing. Separate co-expression networks were constructed for the healed group (*n* = 37) and the non-healed group (*n* = 80) using the Dryad longitudinal bulk RNA-seq cohort. The 2000 genes with the highest variability were selected. The adjacency matrix was established using a threshold of |R| > 0.6 (Pearson correlation matrix; WGCNA R package v1.72). A network analysis was constructed using the igraph R package (v2.2.1). Network cohesion was defined as the arithmetic mean of edge density and the global clustering coefficient. Gene topological importance was assessed via virtual gene knockout (VGK). Cohesion changes were calculated after sequentially removing target nodes. The impact was defined as baseline cohesion minus post-knockout cohesion. The differential impact score (DI) was used to screen for candidate targets exhibiting preferential structural dependence in the non-healed network. The methodology references the scTenifoldKnk framework and is adapted for bulk RNA-seq data (scTenifoldKnk R package V 1.0.3; https://github.com/cailab-tamu/scTenifoldKnk (accessed on 1 March 2026)). For single-cell datasets (GSE165816), a supplementary VGK algorithm was employed using the top 1000 highly variable genes.

In this study, the transport module is functionally defined as a curated gene set covering intracellular vesicle trafficking, endosomal recycling, and cytoskeleton-dependent cargo transport. Genes were drawn from curated Gene Ontology Biological Process terms related to vesicle-mediated transport, endocytic recycling, motor-associated cargo transport, and vesicle targeting (including GO:0016192, GO:0032456, GO:0008089, and GO:0098876), and were filtered for those present in the top 2000 variable genes of the discovery cohort. The module does not include extracellular solute transport, ion-channel transport, or systemic vascular transport. The full gene list is provided in Supplementary Table S1.

### 2.3. Feature Selection in Machine Learning

The transport pathway genes were taken as feature variables and healing outcomes as the outcome variable. Feature selection was performed using the random forest. The 10-fold cross-validation was employed to rank the variables by importance and select the most relevant features. The transport module was used as a pre-defined feature set in order to test the specific hypothesis that transport-related genes carry sufficient information to discriminate healing outcomes. We did not extend the feature set to broader gene panels because the present analysis is hypothesis testing rather than unbiased biomarker discovery; an unrestricted genome-wide search was outside the scope of this study and would be the appropriate next step.

### 2.4. Single-Cell RNA Sequencing Analysis

The GSE165816 single-cell data were processed using Seurat v5.0. Low-quality cells were excluded (nFeature > 200; percent.mt < 15%). After normalizing the data using LogNormalize (scale factor 10,000), 2000 highly variable genes were selected for subsequent analysis. PCA dimensionality reduction and UMAP embedding were performed using standard procedures, and Louvain clustering was executed to identify cell populations. Keratinocyte clusters were identified using KRT14/KRT10/KRT6A module scores. Those with the highest median scores were selected for downstream analysis. Additionally, KIF13A expression patterns were examined in fibroblast, endothelial, and myeloid cell clusters to assess cellular heterogeneity.

Relative immune-cell signature scores were estimated by transcriptomic deconvolution using single-sample gene-set enrichment analysis (ssGSEA) implemented in the GSVA R package (v1.46), based on canonical immune-cell marker gene sets. The output is therefore a computationally inferred immune-cell signature, not a direct measurement of tissue infiltration; histological or spatial validation was not performed. The relevant gene sets (including the immune-cell marker gene sets used here) are listed in Supplementary Table S2.

### 2.5. Virtual Perturbation Analysis

Two types of virtual perturbation analyses were performed in keratinocytes: network collapse analysis and hierarchical comparison analysis. 1000 highly variable genes and known wound-healing effector molecules were determined. After removing the KIF13A node, the redistribution of PageRank centrality was quantified to assess the gene’s contribution to network topology. Cells were grouped based on endogenous KIF13A expression: the high-expression cohort (upper quartile) was termed the “KIF13A-high-expressing” group, and the zero-detection cohort the “KIF13A-low-detection” group. Since zero counts in scRNA-seq may arise from technical dropout, the latter group is interpreted as expression-based stratification rather than experimental gene ablation, and no imputation was applied to avoid introducing artificial gene–gene correlations. Migration phenotype scores were calculated based on the gene set and intergroup differences were compared using the Wilcoxon rank-sum test.

### 2.6. Transcription Factor Correlation Analysis

To identify upstream regulators of KIF13A, the Spearman rank correlation analysis was performed between KIF13A expression in keratinocyte subpopulations and 38 human transcription factors.

### 2.7. Data-Driven Signal Transduction Modeling

Keratinocytes were divided into deciles based on KIF13A expression levels, and the activity of the EGF, PDGF, and FGF pathways in each group was quantified using a gene set scoring method (the mean of Z-standardized log-normalized expression of pathway member genes). The dose-response relationship between KIF13A expression quantiles and pathway activation scores was used to identify potential therapeutic windows. To complement the mechanistic interpretation, a conceptual Hill-equation simulation was used to illustrate the theoretical constraints of transport efficiency on receptor signaling. For all module-based analyses in this study, the inflammation score, EGF/PDGF/FGF pathway activation scores, and migration phenotype score were computed in the same way: as the mean of Z-standardized log-normalized expression of curated marker genes per cell or sample. The full gene lists are provided in Supplementary Table S2. These scores are intended for relative comparison within a dataset and are not direct biochemical measurements of pathway activity.

### 2.8. In Silico Drug Repurposing

To identify potential therapeutic agents for diabetic foot ulcers, this study employed gene set enrichment analysis. The 400 genes showing the most significant differences between non-healed and healed samples (200 upregulated and 200 downregulated) were used as a disease signature. This signature was submitted to the DSigDB and LINCS L1000 drug-perturbation databases (queried via the enrichR interface). The significance of the enrichment was evaluated using Fisher’s exact test with Benjamini–Hochberg multiple-comparison correction (q < 0.05). For the exploratory epalrestat-related simulation (see Section 3.6), the migration module score was recalculated after the disease-associated expression profile was computationally adjusted by subtracting the inferred AKR1B1-related drug-reversal signature, weighted by signature strength. This analysis was treated as exploratory and hypothesis-generating only, and the resulting score is not interpreted as a predicted treatment outcome.

### 2.9. Multi-Cohort Validation

External validation was conducted in the independent bulk DFU cohort GSE134431, which is the most directly comparable independent dataset (thirteen cases of active ulcers vs. eight cases of non-ulcerated diabetic skin). RPKM values were log_2_-transformed and differential expression of KIF13A and other transport-module hub genes (EPN1, CLIP1) was analyzed using limma (v3.56.2) with Benjamini–Hochberg correction. 

### 2.10. Statistical Analysis

All statistical analyses were performed using R v4.3.2. Network analysis was performed using the igraph v2.2.1 and WGCNA v1.72 packages; machine learning models were constructed using the randomForest and glmnet v4.1-8 R packages; and single-cell data processing was performed using the Seurat v5.0 package. Differential expression for the external validation cohort was performed using limma v3.56.2; ssGSEA-based immune-cell signature scoring was performed using the GSVA R package v1.46. Structural equation modeling was performed using the lavaan R package. The Benjamini–Hochberg FDR method was uniformly applied for multiple comparison correction, and a two-sided *p*-value < 0.05 was considered statistically significant.

## 3. Results

### 3.1. Network Topology Identifies Intracellular Transport as the Dominant Vulnerability in Non-Healing Wounds

A total of 117 longitudinal samples (17 patients) were included (37 healed and 80 non-healed, as shown in Table 1). The original study was conducted in accordance with the local institutional review board (IRB) requirements and received ethical approval. For the present secondary analysis of publicly available de-identified data, additional informed consent and institutional ethics approval were not required according to our institutional policy.

**Figure 1:**
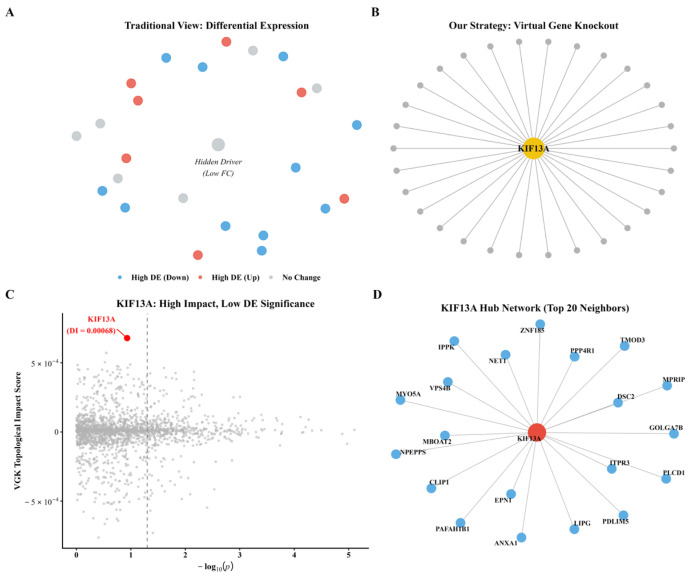
Virtual Gene Knockout Prioritizes KIF13A as a Candidate Topological Network Hub. (**A**) Conventional differential expression analysis picks up high-abundance effectors (red/blue nodes) but misses central drivers with modest fold-changes (gray node). (**B**) VGK identifies KIF13A (gold) by network centrality instead of expression level. (**C**) Driver discovery plot: KIF13A (red dot) sits in the top-left quadrant—high topological impact, insignificant differential expression. The vertical dashed line indicates the nominal significance threshold (*p* = 0.05, −log_10_(*p*) = 1.3). (**D**) KIF13A hub network with top 20 neighbors (EPN1, CLIP1, MYO5A, VPS4B).

The co-expression network revealed that intracellular transport pathways occupy a central topological position within the healing-related network. Gene set enrichment analysis revealed that their functions are concentrated in endosomal dynamics, vesicle-mediated transport, and cytoskeletal organization. Random forest feature importance screening ranked KIF13A seventh among transport-related genes. Paradoxically, while this gene showed no statistical significance in standard differential expression analysis (log_2_FC = 0.173, *p* = 0.263), it ranked first in the virtual gene knockout topological impact score. This contrast highlights the unique value of network analysis methods in identifying hidden key genes (Table 2). The LASSO logistic regression model achieved an AUC of 0.69. The linear mixed-effects model revealed a marginal association between the transport module and healing outcomes (*p* = 0.081). The coefficient of variation for the effect size in leave-one-out cross-validation was 0.14. Figure 1A–C visualizes this pattern, and Figure 1D maps KIF13A’s 20-gene hub neighborhood, including EPN1, CLIP1, MYO5A, and VPS4B.

**Figure 2:**
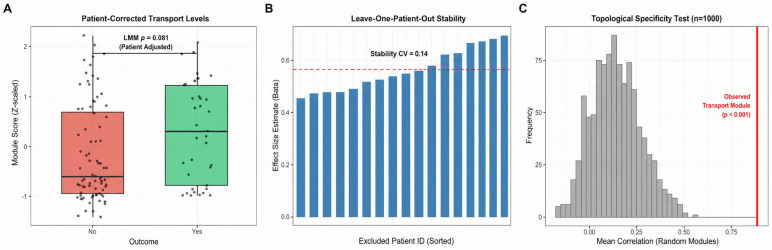
Validation of the Transport Module. (**A**) Transport module scores in healed vs. non-healed wounds, corrected for patient effects. (**B**) Leave-one-patient-out cross-validation: effect sizes remain stable when excluding any single patient (CV = 0.14). (**C**) Permutation test (*n* = 1000 random modules): the transport module’s internal correlation (red line) exceeds random expectations (*p* < 0.001), confirming its co-expression coherence is not due to chance.

**Figure 3:**
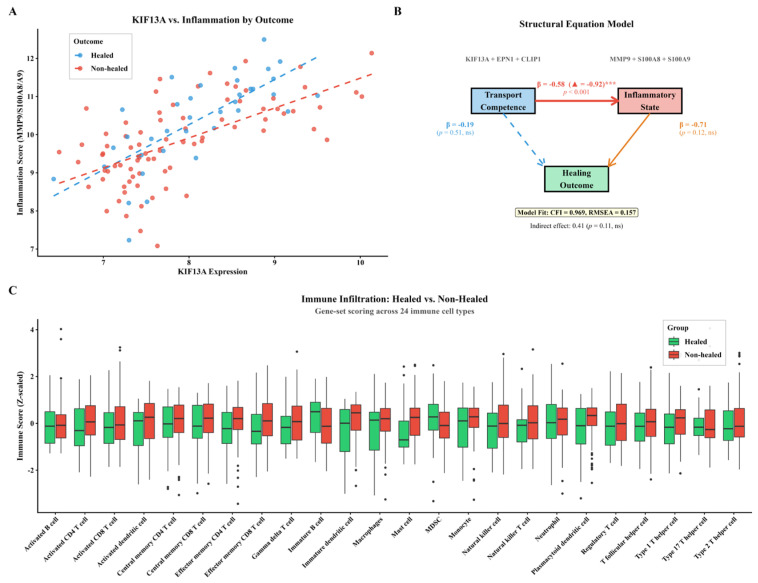
Transport–Inflammation Axis in DFUs. (**A**) KIF13A expression vs. inflammation score (MMP/S100A/cytokine composite; genes listed in Supplementary Table S2). Healed patients (blue) cluster in the high-transport/low-inflammation zone; non-healed patients (red) show high inflammation regardless of KIF13A level. Dashed lines indicate group-specific trends. (**B**) Structural equation model: transport negatively predicts inflammation (unstandardized β = −0.58, standardized β = −0.92, *p* < 0.001), with inflammation trending toward worse outcome (unstandardized β = −0.71, *p* = 0.12); the direct transport→outcome path is weak (unstandardized β = −0.19, *p* = 0.51). Path coefficients shown in the diagram are unstandardized estimates from the lavaan SEM. Model fit is adequate (CFI = 0.97, SRMR = 0.046) despite elevated RMSEA due to limited sample size. The dashed blue arrow represents the direct path from Transport Competence to Healing Outcome; solid arrows represent the paths of the indirect (mediated) pathway. “▲ standardized β; ***, *p* < 0.001; ns, not significant.” (**C**) Computationally inferred immune-cell signatures (ssGSEA via GSVA on bulk transcriptomes; not direct histological measurement of infiltration) in healed (green) vs. non-healed (red) wounds. Activated dendritic cells (R = −0.58), regulatory T-cells (R = −0.54), and macrophages (R = −0.53) correlate most strongly with low KIF13A.

Module-level validation held across patient adjustment, resampling, and a topological specificity test against random gene sets (Figure 2). Mechanistically, the transport module sat upstream of inflammation: KIF13A correlated with inflammatory load in both groups but healed patients occupied a high-transport/low-inflammation zone, structural equation modeling placed inflammation as the dominant mediator between transport and outcome, and activated dendritic cells, regulatory T cells, and macrophages were the immune populations most anti-correlated with KIF13A (Figure 3).

**Figure 4:**
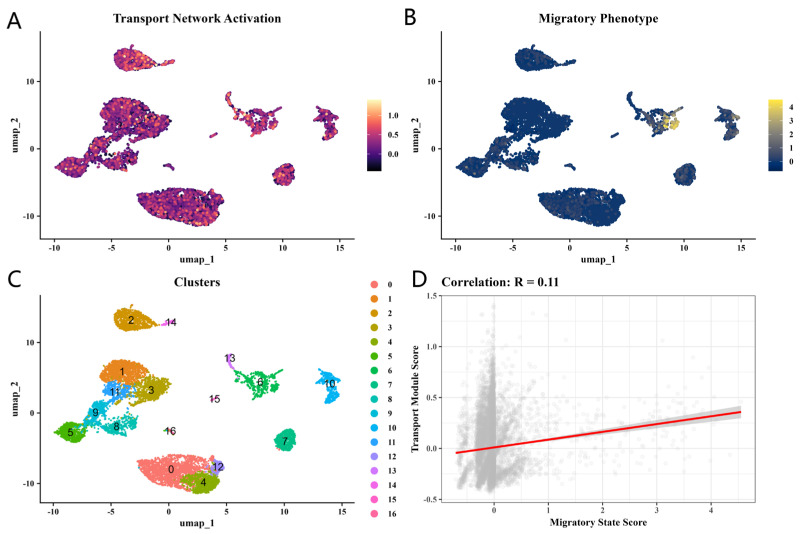
Single-cell transcriptomic validation of transport network activation. (**A**) UMAP visualization of transport network activation scores across all cell clusters in GSE165816. (**B**) UMAP of migratory phenotype scores. (**C**) Louvain clustering identified 17 cell populations (clusters 0–16). (**D**) Single-cell correlation between the transport module score and migratory state score (R = 0.11), demonstrating a positive but heterogeneous association. The red line in panel (**D**) indicates the linear regression trend line (Pearson R = 0.11).

**Figure 5:**
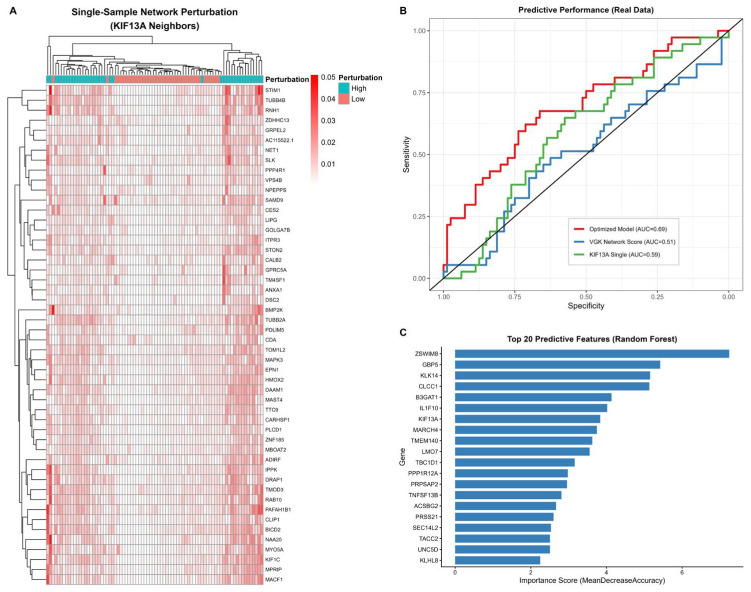
Patient Stratification and Feature Screening. (**A**) Single-sample network perturbation of KIF13A neighbors in non-healing patients, identifying high-disruption (cyan) and low-disruption (salmon) subtypes. (**B**) ROC curves for predictive models: multi-gene model (AUC = 0.69, red) outperforms VGK network score (AUC = 0.51, blue dashed) and KIF13A alone (AUC = 0.59, green dotted). The black diagonal line in panel (**B**) indicates the reference line for random classification (AUC = 0.50). Models were used for feature screening given limited sample size (*n* = 17 patients, 117 observations). (**C**) Top 20 predictive features by random forest importance, with ZSWIM8, GBP5, KLK14, and KIF13A ranked highest.

At single-cell resolution, transport network activity was broadly distributed across cell types while the migratory phenotype concentrated in a smaller subset, with only modest per-cell coupling between the two (Figure 4). At the patient scale, single-sample perturbation of the KIF13A neighborhood further stratified non-healing patients into high-disruption and low-disruption subtypes (Figure 5).

### 3.2. Virtual Gene Knockout Prioritizes KIF13A as a Candidate Load-Bearing Network Hub

**Figure 6:**
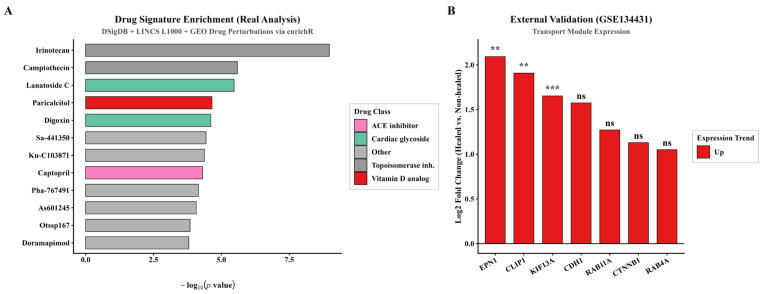
Drug Repurposing and External Validation. (**A**) Drug signature enrichment (DSigDB and LINCS L1000 via enrichR) using the top 200 up- and 200 down-regulated genes from non-healing vs. healing DFUs. Paricalcitol (vitamin D analog; rank 6; adjusted *p* = 0.036) was among the significantly enriched disease-reversing (DOWN-direction) signatures and was prioritized for biological relevance to wound inflammation. AKR1B1 appeared recurrently across drug-related signatures, nominating the aldose-reductase pathway; epalrestat was prioritized as a hypothesis-generating AKR1B1 inhibitor rather than a direct top-ranked enrichment hit. (**B**) External validation (GSE134431): transport module genes in DFUs vs. non-ulcerated diabetic skin. Asterisks indicate Benjamini–Hochberg adjusted *p* values: KIF13A, EPN1, and CLIP1 were significantly differentially expressed. ***, *p* < 0.001; **, *p* < 0.01; ns, not significant.

Virtual gene knockouts were performed in both bulk (constructed from the Dryad longitudinal cohort) and single-cell co-expression networks. Bulk network analysis suggested that computational removal of KIF13A had a disproportionate topological impact on the transport–inflammation axis: in a structural-equation model, the standardized path coefficient for the transport→inflammation pathway was β = −0.92 (*p* < 0.001), with good model fit (CFI = 0.97). However, the direct pathway from transport to healing outcomes was not statistically significant (β = −0.19, *p* = 0.51). The indirect effect (transport → inflammation → outcome) was *p* = 0.116, and a causal mediation relationship is proposed only as a hypothesis (Figure 2 and Figure 3). In the single-cell keratinocyte network, computational removal of the KIF13A node was associated with widespread redistribution of centrality. Drug-repurposing enrichment results and independent cohort validation are presented in Figure 6 and Table 3.

Table 3 lists the top enriched and therapeutically relevant compounds; the full ranked list will be made available in the code repository.

**Table 3:** Drug signature enrichment analysis results (top 30 hits by *p*-value, plus therapeutically relevant compounds).

Rank	Drug/Compound	Database	Signature Direction	Overlap	*p* Value	Adjusted *p*
1	Irinotecan	DSigDB	DOWN	38/1272	1.05 × 10^−9^	1.46 × 10^−6^
2	Irinotecan	DSigDB	DOWN	30/999	6.92 × 10^−8^	4.80 × 10^−5^
3	Camptothecin	DSigDB	DOWN	35/1513	2.56 × 10^−6^	0.0012
4	Lanatoside C	DSigDB	UP	16/405	3.38 × 10^−6^	0.0012
5	Neostigmine bromide	DSigDB	DOWN	20/650	8.82 × 10^−6^	0.0024
6	Paricalcitol	DSigDB	DOWN	6/57	2.20 × 10^−5^	0.0359
7	Digoxin	DSigDB	UP	13/324	2.44 × 10^−5^	0.0054
8	Sanguinarine	DSigDB	DOWN	14/376	2.71 × 10^−5^	0.0054
9	SA-441350	DSigDB	DOWN	11/244	3.65 × 10^−5^	0.1403
10	KU-C103871	DSigDB	UP	11/248	4.23 × 10^−5^	0.1403
11	Captopril	DSigDB	DOWN	22/856	4.94 × 10^−5^	0.0086
12	Helveticoside	DSigDB	UP	13/355	6.25 × 10^−5^	0.0095
13	PHA-767491	LINCS L1000	DOWN	7/101	7.00 × 10^−5^	0.0095
14	Anisomycin	DSigDB	UP	26/1142	7.57 × 10^−5^	0.0095
15	PHOSPHINE	DSigDB	DOWN	6/71	7.74 × 10^−5^	0.0630
16	AS601245	LINCS L1000	DOWN	5/45	8.40 × 10^−5^	0.0097
17	Lobeline	DSigDB	DOWN	31/1510	1.01 × 10^−4^	0.0105
18	Acetaminophen	DSigDB	DOWN	64/4135	1.08 × 10^−4^	0.0105
19	Proscillaridin	DSigDB	UP	13/378	1.17 × 10^−4^	0.0105
20	Cephaeline	DSigDB	DOWN	10/234	1.28 × 10^−4^	0.0105
21	Deptropine	DSigDB	DOWN	14/435	1.29 × 10^−4^	0.0105
22	OTSSP167	LINCS L1000	DOWN	10/237	1.42 × 10^−4^	0.0110
23	LY-294002	DSigDB	UP	10/240	1.58 × 10^−4^	0.1403
24	Doramapimod	DSigDB	DOWN	10/240	1.58 × 10^−4^	0.1403
25	LY-303511	DSigDB	UP	10/242	1.69 × 10^−4^	0.1403
26	METHYL METHANESULFONATE	DSigDB	DOWN	60/3864	1.80 × 10^−4^	0.0132
27	L-755507	DSigDB	DOWN	10/244	1.80 × 10^−4^	0.4279
28	ARG-CSC-26	DSigDB	DOWN	10/244	1.80 × 10^−4^	0.1403
29	SA-90544	DSigDB	DOWN	10/244	1.80 × 10^−4^	0.1403
30	Kifunensine	DSigDB	DOWN	10/245	1.87 × 10^−4^	0.4279
31 †	Ibuprofen	DSigDB	DOWN	7/149	7.63 × 10^−4^	0.3107
32 †	PD 98059	DSigDB	DOWN	9/262	1.33 × 10^−3^	0.3930
33 †	Dinoprostone	DSigDB	DOWN	6/127	1.76 × 10^−3^	0.3930

Drug signature enrichment performed via enrichR against DSigDB and L1000. Disease signature: top 200 up- and down-regulated genes in non-healing vs. healing DFUs (ranked by limma t-statistic). *p* values: Fisher’s exact test; adjusted: Benjamini–Hochberg. Signature direction: UP = drug mimics disease; DOWN = drug reverses disease (therapeutic). Overlap: shared genes/total in drug set. † Rows 31–33: additional compounds discussed in text (ranks 61, 94, 104/400). Epalrestat not a direct hit; selected via AKR1B1 recurrence across signatures and prior wound healing evidence. Full results (400 entries) will be deposited in the code repository upon acceptance.

### 3.3. Single-Cell Expression Stratification Links KIF13A to Migratory Competence

**Figure 7:**
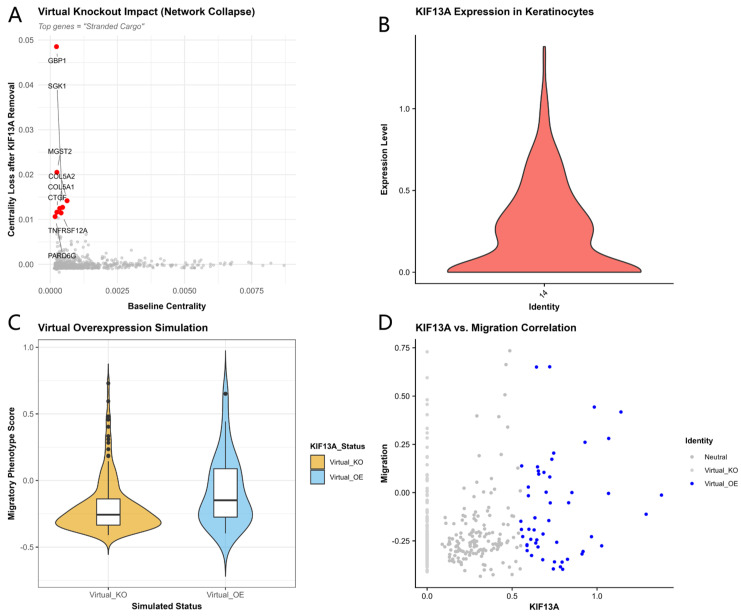
Single-cell virtual perturbation analysis of KIF13A in wound-edge keratinocytes. (**A**) Virtual knockout impact plot showing centrality loss after KIF13A removal. Top “stranded cargo” genes (GBP1, MGST2, COL5A2, CTGF) are highlighted in red. (**B**) Violin plot of KIF13A expression distribution in the keratinocyte cluster. (**C**) Expression stratification analysis: comparison of migratory phenotype scores between KIF13A-low-detection (zero detected transcripts) and KIF13A-high-expressing (top 25%) keratinocytes (Wilcoxon *p* = 0.0058). (**D**) Scatter plot of KIF13A expression versus migration score, colored by group assignment.

Virtual knockout simulation of KIF13A within the single-cell keratinocyte co-expression network identified a set of genes—including GBP1, MGST2, CTGF, and COL5A2—that exhibited the greatest loss of network centrality upon KIF13A removal, designating them as KIF13A-dependent network effectors (Figure 7A). The distribution of KIF13A expression in keratinocytes exhibited marked intercellular heterogeneity, with a large fraction of cells having no detected KIF13A transcripts and a smaller subset showing higher expression (Figure 7B). The top 25% of high-expressing cells were assigned to the “KIF13A-high-expressing” group, while cells with zero detected KIF13A transcripts were assigned to the “KIF13A-low-detection” group. The migration phenotype score was significantly higher in the high-expression group compared to the zero-detection group (Wilcoxon rank-sum test, *p* = 0.0058; Figure 7C). Scatter plot analysis revealed a positive correlation between the two, with high-expressing cells clustering in the high-migration quadrant (Figure 7D).

### 3.4. Upstream Regulatory Analysis Identifies Candidate Transcriptional Regulators of KIF13A

**Figure 8:**
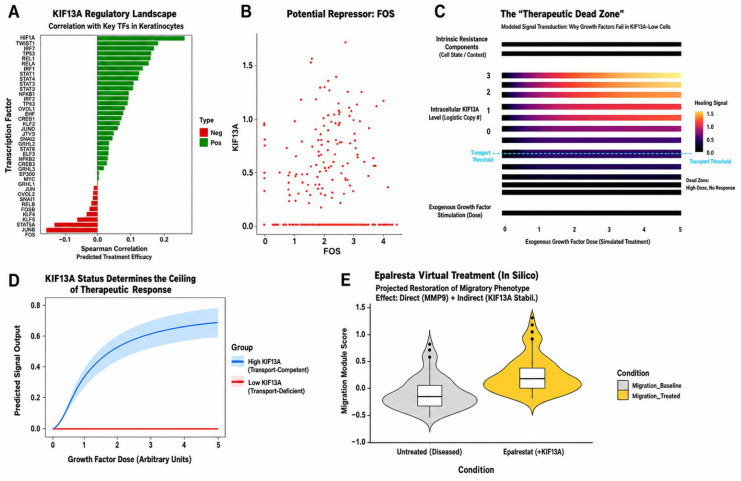
Upstream regulation, signal transduction modeling, and therapeutic simulation. (**A**) Transcription factor regulatory landscape: Spearman correlation of KIF13A with 38 transcription factors in keratinocytes. HIF1A is the top positive correlator (ρ = 0.26); FOS is the most significant negative correlator (ρ = −0.16). (**B**) Scatter plot of FOS versus KIF13A expression at single-cell resolution. (**C**) Conceptual Hill-equation simulation showing how reduced transport capacity is predicted to constrain growth-factor responsiveness; the panel is conceptual and is not derived from experimental dose-response measurements. (**D**) Stratified Hill-equation simulation: transport-competent cells (blue) produce a sigmoidal response, whereas transport-deficient cells (red) are constrained to baseline. The simulation is conceptual and not derived from measured dose-response data. (**E**) Exploratory epalrestat-related simulation: violin plots comparing inferred migration module scores between untreated diseased keratinocytes and cells in which the disease-associated expression profile was computationally adjusted using the inferred drug-reversal signature. This panel is hypothesis-generating only; see Section 2.8 for details.

The hypoxia-inducible factor HIF1A showed the strongest positive correlation (ρ = 0.26, *p* < 0.001) (Figure 8A). FOS showed a significant negative correlation with KIF13A at single-cell resolution (ρ = −0.16, *p* < 0.001; Figure 8B), and this association was also observed in the bulk longitudinal cohort (*n* = 117 samples; ρ = −0.32, *p* < 0.001). Other members of the AP-1 family, JUNB and FOSB, also exhibited consistent negative trends (Figure 8A).

### 3.5. Data-Driven Modeling Reveals a Transport-Dependent Threshold for Growth Factor Responsiveness

Keratinocytes were divided into ten groups based on KIF13A expression, and the activation levels of growth factor pathways were calculated. The dose-response relationship exhibited a clear threshold effect: when KIF13A expression was below the 30th percentile, activation scores for the EGF, PDGF, and FGF pathways remained near baseline levels; beyond this threshold, scores increased in a dose-dependent manner as KIF13A expression rose (Figure 8C). A Hill-equation simulation was used to make the implication of this threshold pattern explicit at a conceptual level: transport-competent cells were represented as producing a sigmoidal response, whereas transport-deficient cells, with maximal response constrained to baseline, were represented as producing a flat profile (Figure 8D). The Hill simulation is conceptual and is not derived from experimental dose-response measurements.

### 3.6. In Silico Drug Repurposing Identifies Epalrestat as a Candidate Transport-Restoring Agent

Based on a transport dysfunction model, this study screened the DSigDB and LINCS L1000 drug-perturbation databases. Among the significantly enriched disease-reversing (DOWN-direction) signatures, paricalcitol (a vitamin D analog; adj. *p* = 0.036) was a notable hit, consistent with evidence that vitamin D regulates wound inflammation. AKR1B1 appeared recurrently across multiple drug-related signatures, nominating the aldose-reductase pathway. Epalrestat, a clinically used AKR1B1 inhibitor, was therefore prioritized as a hypothesis-generating candidate compound rather than as a direct top-ranked enrichment hit (Figure 6A and Figure 8E).

### 3.7. Multi-Cohort Validation Confirms Transport Gene Dysregulation in DFU Tissue

In the independent bulk validation cohort GSE134431 (DFUs vs. non-ulcerated diabetic skin), KIF13A (log_2_FC = 0.659; Fold-Change = 1.58; adj. *p* = 0.0075), EPN1 (log_2_FC = 1.024; Fold-Change = 2.03; adj. *p* = 0.0204), and CLIP1 (log_2_FC = 0.901; Fold-Change = 1.87; adj. *p* = 0.0168) were all significantly upregulated (Figure 6B; full statistics in Table 4). Although KIF13A expression is elevated at the population level, a subset of cells still expresses KIF13A below the functional threshold required for effective signal transduction.

## 4. Discussion

Diabetic foot ulcers show marked heterogeneity in both cellular behavior and treatment response [15,16,17,18]. Growth factor supplementation is widely used, yet many patients fail to heal even after adequate dose [18,19]. The mechanisms underlying this resistance remain poorly understood.

Our findings suggest a specific hypothesis. KIF13A-associated vesicle transport impairment may act as a candidate cellular bottleneck in a subset of DFU cells. Under this model, impaired intracellular vesicle transport could reduce receptor trafficking or signaling responsiveness. Exogenous growth factors may therefore be present, yet cellular responsiveness may remain limited. This interpretation remains inferential and would require direct testing by receptor-localization and perturbation experiments.

Kinesin motor proteins drive anterograde vesicle movement along microtubule tracks. They regulate membrane trafficking, organelle positioning, and cell polarity [20]. KIF13A belongs to the kinesin-3 subfamily and functions as a structural network hub [21,22,23]. It mediates peripheral endosome transport and influences the recycling and signaling output of membrane receptors, including receptor tyrosine kinases. Its precise role in EGFR trafficking remains to be fully defined [22]. Our data reveal a threshold effect. Below the 30th percentile of KIF13A expression, EGF, PDGF, and FGF pathway activation scores remain near baseline. Only above this threshold does signaling increase in a dose-dependent manner. A minimum level of transport capacity is therefore required for effective growth factor signal transduction.

This threshold model implies that DFUs may include two distinct subtypes: transport-competent and transport-deficient. In transport-deficient cells, adding more growth factor cannot overcome the intracellular bottleneck. Future clinical trials should consider stratifying patients by transport module status and testing interventions that restore vesicle transport, rather than simply escalating growth factor doses.

KIF13A was upregulated at the population level in DFU tissues. This seems counterintuitive if transport failure contributes to non-healing. Single-cell data provide a possible explanation. Upregulation in some cells may reflect a compensatory response, whereas other cells show absent or low-detected KIF13A expression below the inferred functional threshold. These low-detection cells may represent a candidate cellular bottleneck. Population-level averages may obscure this subgroup. This “masking effect” highlights why bulk expression analysis is insufficient for understanding complex wound dysfunction.

Drug enrichment analysis nominated the aldose-reductase pathway, and epalrestat was prioritized as a hypothesis-generating AKR1B1 inhibitor. Hyperglycemia activates the polyol pathway, which may perturb cytoskeletal dynamics and microtubule-dependent transport. Because KIF13A function depends on intact microtubule tracks, we hypothesize that targeting AKR1B1 could help preserve transport capacity under diabetic stress conditions. This possibility requires direct experimental validation.

Several questions remain. First, it is unclear whether downstream effectors—GBP1, COL5A2, and CTGF—are genuinely regulated by KIF13A transport activity. This needs direct experimental testing. Second, whether epalrestat can rescue their aberrant expression requires validation in cell or animal models. Third, spatial mapping of KIF13A expression gradients on tissue sections would clarify whether functional uncoupling concentrates in specific wound regions. Fourth, live-cell imaging probes or selective KIF13A inhibitors would allow real-time observation of transport dynamics. Finally, a clinical trial targeting the transport module is needed to assess translational value.

This study has several limitations. Because all analyses are based on secondary transcriptomic datasets and computational network perturbation, the findings should be interpreted as evidence of network vulnerability and functional association rather than proof of causal necessity; direct perturbation experiments such as CRISPR-mediated KIF13A knockdown and rescue in keratinocyte wound-closure models will be required. First, network centrality estimates and module scores may be influenced by unmodeled biological or technical confounders, including cell-cycle phase, metabolic activity, transcriptional bursting, sequencing depth, and cell-type composition; although normalization and variable-gene filtering were applied, these factors cannot be fully excluded. Second, zero counts in scRNA-seq may reflect technical dropout, so the KIF13A-low-detection group should be interpreted as expression-based stratification rather than a true genetic knockout; no imputation was applied in the primary analysis to avoid introducing artificial gene–gene correlations. Third, the immune-cell results were computationally inferred from transcriptomic signatures via ssGSEA and were not confirmed by histology, immunohistochemistry, or spatial profiling. Fourth, the sample size is small and predictive models should be interpreted with caution. Fifth, the upstream mechanisms suppressing KIF13A in deficient cells, including transcriptional repression and epigenetic regulation, remain unexplored. Finally, the proposed therapeutic relevance of epalrestat is hypothesis-generating and requires validation in cellular, animal, and clinical studies.

## 5. Conclusions

This secondary computational analysis identifies KIF13A-associated intracellular vesicle transport impairment as a candidate systems-level vulnerability in chronic DFUs. The results suggest that impaired transport capacity may limit growth-factor pathway responsiveness in a subset of cells, even when growth-factor signals are present. Because the present study is observational and computational, these findings should be interpreted as hypothesis-generating. Epalrestat, identified through drug-signature enrichment via recurrent AKR1B1 hits, is presented as a candidate compound for experimental testing. Direct perturbation studies and prospective clinical validation are required to determine whether restoring vesicle transport can improve wound healing.

## Figures and Tables

**Table 1:** Baseline characteristics of the longitudinal DFU cohort.

Characteristic	Healed Group (*n* = 37 Samples)	Non-Healed Group (*n* = 80 Samples)	*p* Value
Study design	Longitudinal wound-edge RNA-seq		
Patients (*n*)	9	8	
Total samples (*n*)	37	80	
Samples per patient, median (range)	4 (2–6)	10 (5–13)	
Time of biopsy, weeks, mean (SD)	3.5 (2.0)	7.5 (4.7)	1.28 × 10^−9^
Healing outcome	Complete closure ≤ 12 weeks	Non-closure at 12 weeks	
Sequencing platform	RNA-seq (CogentAP/STAR)	RNA-seq (CogentAP/STAR)	

**Table 2:** Comparison of top regulatory drivers identified by standard DEG analysis and virtual gene knockout (VGK). VGK ranks genes by differential impact (DI) score on network cohesion; logFC and *p* values from DEG are shown for comparison to demonstrate that top VGK drivers are invisible to standard DE.

Method	Rank	Gene	LogFC/DI Score	*p* Value
Standard DEG (*p*-value)	1	AC022167.4	−0.590	<0.001
	2	TNFRSF11B	−1.978	<0.001
	3	ADTRP	−0.819	<0.001
	4	RCN1P2	−1.427	<0.001
	5	CX3CL1	−1.093	<0.001
	6	SIK1	1.844	<0.001
	7	STC2	−1.543	<0.001
	8	MTCYBP18	−1.372	<0.001
	9	CEP85	−0.577	<0.001
	10	MTND3P19	−0.449	<0.001
VGK (DI Score)	1	KIF13A	0.000679	0.263
	2	GPRC5A	0.000572	ns
	3	ABCC8	0.000504	ns
	4	SPTBN4	0.000488	ns
	5	UNC5D	0.000484	ns
	6	MARCH4	0.000476	ns
	7	SLC12A1	0.000472	ns
	8	MYBPC2	0.000465	ns
	9	B3GAT1	0.000464	ns
	10	PRSS21	0.000455	ns

Abbreviations: DEG, Differentially Expressed Gene; VGK, Virtual Gene Knockout; DI, Differential Impact; LogFC, Log_2_ Fold Change; ns, not significant (*p* > 0.05). For DEG (top) rows, the values shown are LogFC and DEG *p* value from limma differential expression. For VGK (bottom) rows, the values shown in the LogFC/DI score column are the differential impact (DI) score, calculated as Impact_nonhealed−Impact_healed; the values in the *p*-value column are the corresponding DEG significance from limma, illustrating that several VGK-prioritized network drivers escape standard differential expression detection. For example, KIF13A is the top VGK hit (DI = 0.000679) but is not differentially expressed (log_2_FC = 0.173, *p* = 0.263).

**Table 4:** Differential expression of transport-module hub genes in the independent bulk validation cohort GSE134431 (DFUs vs. non-ulcerated diabetic skin; limma).

Gene	log_2_FC	Fold-Change	t-Statistic	*p*-Value (Raw)	adj. *p* (BH)	Direction
KIF13A	0.659	1.58	4.108	0.00057	0.0075	Up
EPN1	1.024	2.03	3.457	0.00257	0.0204	Up
CLIP1	0.901	1.87	3.577	0.00195	0.0168	Up

## Data Availability

The primary longitudinal DFU transcriptomic dataset is available from the Dryad Digital Repository (https://doi.org/10.5061/dryad.2v6wwpzzc), with raw sequencing data deposited in the NCBI Sequence Read Archive (PRJNA1200081). Single-cell RNA sequencing data (GSE165816) and the external validation dataset (GSE134431) are available from the NCBI Gene Expression Omnibus (https://www.ncbi.nlm.nih.gov/geo/ (accessed on 1 March 2026)). All analysis scripts will be deposited in a public GitHub repository (https://github.com/rht888666/DFU_KIF13A_Network (accessed on 1 March 2026)) upon acceptance to support reproducibility. All statistical analyses were performed in R v4.3.2 (R Foundation for Statistical Computing, Vienna, Austria). Co-expression network analysis used the WGCNA (v1.72) [24] and igraph (v2.2.1) [25] packages. Single-cell data were processed with Seurat (v5.0) [26]. Immune-cell signature scoring used GSVA (v1.46) [27]. Differential expression analysis used limma (v3.56.2) [28]. Structural equation modeling used lavaan [29]. Machine learning models were built with randomForest [30] and glmnet (v4.1-8) [31].

## Supplementary Materials

The following supporting information can be downloaded at: https://www.mdpi.com/article/10.3390/biomedicines14051140/s1, Table S1: Transport module gene list (*n* = 119); Table S2: Gene sets used for pathway activation scoring and immune-cell signature deconvolution.
