# Supplementary material for: Intracellular Vesicle Transport Impairment as a Candidate Systems-Level Bottleneck in Chronic Diabetic Foot Ulcers: Network Medicine Identifies KIF13A as a Potential Therapeutic Vulnerability
Source: Biomedicines. 2026 May 18;14(5):1140. doi: 10.3390/biomedicines14051140 (PMC13204274; doi:10.3390/biomedicines14051140)
Supplement: Supplementary file 1 [file biomedicines-14-01140-s001.zip › biomedicines-4284549-supplementary.pdf]

Supplementary Materials

Intracellular Vesicle Transport Impairment as a Candidate Systems-Level Bottleneck in Chronic Diabetic Foot Ulcers: Network Medicine Identifies KIF13A as a Potential Therapeutic Vulnerability

Haitao Ren and Yongan Xu

Supplementary Table S1. Transport module gene list (n=119). Genes were derived from curated Gene Ontology Biological Process terms related to vesicle-mediated transport, endocytic recycling, motor-associated cargo transport, and vesicle targeting (GO:0016192, GO:0032456, GO:0008089, and GO:0098876), filtered for those present in the top 2,000 variable genes of the longitudinal DFU cohort.

Gene Symbol	Source GO ID	Functional Category
KIF13A	GO:0016192	Vesicle-mediated transport
KIF13B	GO:0016192	Vesicle-mediated transport
KIF1B	GO:0016192	Vesicle-mediated transport
KIF1C	GO:0016192	Vesicle-mediated transport
KIF3A	GO:0016192	Vesicle-mediated transport
KIF5B	GO:0016192	Vesicle-mediated transport
KIF5C	GO:0016192	Vesicle-mediated transport
MYO5A	GO:0016192	Vesicle-mediated transport
MYO5B	GO:0016192	Vesicle-mediated transport
MYO6	GO:0016192	Vesicle-mediated transport
MYO7A	GO:0016192	Vesicle-mediated transport
RAB4A	GO:0016192	Vesicle-mediated transport
RAB5A	GO:0016192	Vesicle-mediated transport
RAB5B	GO:0016192	Vesicle-mediated transport
RAB5C	GO:0016192	Vesicle-mediated transport
RAB7A	GO:0016192	Vesicle-mediated transport
RAB7B	GO:0016192	Vesicle-mediated transport
RAB11A	GO:0016192	Vesicle-mediated transport
RAB11B	GO:0016192	Vesicle-mediated transport
RAB14	GO:0016192	Vesicle-mediated transport
RAB27A	GO:0016192	Vesicle-mediated transport
RAB27B	GO:0016192	Vesicle-mediated transport
RAB35	GO:0016192	Vesicle-mediated transport
VPS4A	GO:0016192	Vesicle-mediated transport
VPS4B	GO:0016192	Vesicle-mediated transport
VPS26A	GO:0016192	Vesicle-mediated transport
VPS26B	GO:0016192	Vesicle-mediated transport
VPS29	GO:0016192	Vesicle-mediated transport
VPS35	GO:0016192	Vesicle-mediated transport
HRS	GO:0016192	Vesicle-mediated transport
TSG101	GO:0016192	Vesicle-mediated transport
ALIX	GO:0016192	Vesicle-mediated transport
CHMP2A	GO:0016192	Vesicle-mediated transport
CHMP2B	GO:0016192	Vesicle-mediated transport
CHMP3	GO:0016192	Vesicle-mediated transport
CHMP4A	GO:0016192	Vesicle-mediated transport
CHMP4B	GO:0016192	Vesicle-mediated transport
EPN1	GO:0032456	Endocytic recycling
EPN2	GO:0032456	Endocytic recycling
EPN3	GO:0032456	Endocytic recycling
EHD1	GO:0032456	Endocytic recycling
EHD3	GO:0032456	Endocytic recycling
EHD4	GO:0032456	Endocytic recycling
CLIP1	GO:0032456	Endocytic recycling
CLIP2	GO:0032456	Endocytic recycling

CLASP1	GO:0032456	Endocytic recycling
CLASP2	GO:0032456	Endocytic recycling
SNX1	GO:0032456	Endocytic recycling
SNX2	GO:0032456	Endocytic recycling
SNX3	GO:0032456	Endocytic recycling
SNX6	GO:0032456	Endocytic recycling
SNX27	GO:0032456	Endocytic recycling
ACAP1	GO:0032456	Endocytic recycling
ACAP2	GO:0032456	Endocytic recycling
ARFIP2	GO:0032456	Endocytic recycling
TBC1D5	GO:0032456	Endocytic recycling
APP	GO:0008089	Motor-associated cargo transport
SNAP25	GO:0008089	Motor-associated cargo transport
VAMP2	GO:0008089	Motor-associated cargo transport
STX1A	GO:0008089	Motor-associated cargo transport
STXBP1	GO:0008089	Motor-associated cargo transport
DYNLL1	GO:0008089	Motor-associated cargo transport
DYNLL2	GO:0008089	Motor-associated cargo transport
DYNLRB1	GO:0008089	Motor-associated cargo transport
DYNLRB2	GO:0008089	Motor-associated cargo transport
DYNC1H1	GO:0008089	Motor-associated cargo transport
DYNC1I1	GO:0008089	Motor-associated cargo transport
DYNC1I2	GO:0008089	Motor-associated cargo transport
VPS13A	GO:0098876	Vesicle/endosomal targeting
VPS13B	GO:0098876	Vesicle/endosomal targeting
VPS13C	GO:0098876	Vesicle/endosomal targeting
VPS13D	GO:0098876	Vesicle/endosomal targeting
PIKFYVE	GO:0098876	Vesicle/endosomal targeting
MTMR3	GO:0098876	Vesicle/endosomal targeting
MTMR4	GO:0098876	Vesicle/endosomal targeting
FIG4	GO:0098876	Vesicle/endosomal targeting
RAB10	GO:0098876	Vesicle/endosomal targeting
RAB18	GO:0098876	Vesicle/endosomal targeting
RAB22A	GO:0098876	Vesicle/endosomal targeting
RAB23	GO:0098876	Vesicle/endosomal targeting
RAB25	GO:0098876	Vesicle/endosomal targeting
RAB33B	GO:0098876	Vesicle/endosomal targeting
DCTN1	GO:0098876	Vesicle/endosomal targeting
DCTN2	GO:0098876	Vesicle/endosomal targeting
DCTN4	GO:0098876	Vesicle/endosomal targeting
DCTN5	GO:0098876	Vesicle/endosomal targeting
DCTN6	GO:0098876	Vesicle/endosomal targeting
HOOK1	GO:0098876	Vesicle/endosomal targeting
HOOK2	GO:0098876	Vesicle/endosomal targeting
HOOK3	GO:0098876	Vesicle/endosomal targeting
RILP	GO:0098876	Vesicle/endosomal targeting
ORP1	GO:0098876	Vesicle/endosomal targeting
MYO1C	GO:0098876	Vesicle/endosomal targeting
MYO1E	GO:0098876	Vesicle/endosomal targeting
MYO18A	GO:0098876	Vesicle/endosomal targeting
ARPC1A	GO:0098876	Vesicle/endosomal targeting
ARPC1B	GO:0098876	Vesicle/endosomal targeting
ARPC2	GO:0098876	Vesicle/endosomal targeting
ARPC3	GO:0098876	Vesicle/endosomal targeting
ARPC5	GO:0098876	Vesicle/endosomal targeting
NSF	GO:0098876	Vesicle/endosomal targeting
NSFL1C	GO:0098876	Vesicle/endosomal targeting
VCP	GO:0098876	Vesicle/endosomal targeting
VCPIP1	GO:0098876	Vesicle/endosomal targeting
ATPIF1	GO:0098876	Vesicle/endosomal targeting
SEC13	GO:0098876	Vesicle/endosomal targeting
SEC16A	GO:0098876	Vesicle/endosomal targeting

SEC23A	GO:0098876	Vesicle/endosomal targeting
SEC24B	GO:0098876	Vesicle/endosomal targeting
SEC31A	GO:0098876	Vesicle/endosomal targeting
SAR1A	GO:0098876	Vesicle/endosomal targeting
SAR1B	GO:0098876	Vesicle/endosomal targeting
ARF1	GO:0098876	Vesicle/endosomal targeting
ARF3	GO:0098876	Vesicle/endosomal targeting
ARF4	GO:0098876	Vesicle/endosomal targeting
ARF6	GO:0098876	Vesicle/endosomal targeting
GBF1	GO:0098876	Vesicle/endosomal targeting
BIG1	GO:0098876	Vesicle/endosomal targeting
BIG2	GO:0098876	Vesicle/endosomal targeting

Supplementary Table S2. Gene sets used for pathway activation scoring. Each score is computed as the mean Z-standardized log-normalized expression of the listed genes across cells or samples.

Pathway/Score Name	Gene Symbol	Scoring Method
Inflammation Score (MMP/S100A/cytokine composite)	MMP9	Mean Z-standardized log-normalized expression of pathway member genes
Inflammation Score (MMP/S100A/cytokine composite)	S100A8	Mean Z-standardized log-normalized expression of pathway member genes
Inflammation Score (MMP/S100A/cytokine composite)	S100A9	Mean Z-standardized log-normalized expression of pathway member genes
Inflammation Score (MMP/S100A/cytokine composite)	IL1B	Mean Z-standardized log-normalized expression of pathway member genes
Inflammation Score (MMP/S100A/cytokine composite)	TNF	Mean Z-standardized log-normalized expression of pathway member genes
Inflammation Score (MMP/S100A/cytokine composite)	CXCL8	Mean Z-standardized log-normalized expression of pathway member genes
Inflammation Score (MMP/S100A/cytokine composite)	IL6	Mean Z-standardized log-normalized expression of pathway member genes
Inflammation Score (MMP/S100A/cytokine composite)	MMP1	Mean Z-standardized log-normalized expression of pathway member genes
Inflammation Score (MMP/S100A/cytokine composite)	MMP3	Mean Z-standardized log-normalized expression of pathway member genes
EGF Pathway	EGFR	Mean Z-standardized log-normalized expression of pathway member genes
EGF Pathway	EGF	Mean Z-standardized log-normalized expression of pathway member genes
EGF Pathway	ERBB2	Mean Z-standardized log-normalized expression of pathway member genes
EGF Pathway	ERBB3	Mean Z-standardized log-normalized expression of pathway member genes
EGF Pathway	GRB2	Mean Z-standardized log-normalized expression of pathway member genes
EGF Pathway	SOS1	Mean Z-standardized log-normalized expression of pathway member genes
EGF Pathway	KRAS	Mean Z-standardized log-normalized expression of pathway member genes
EGF Pathway	BRAF	Mean Z-standardized log-normalized expression of pathway member genes
EGF Pathway	MAP2K1	Mean Z-standardized log-normalized expression of pathway member genes
EGF Pathway	MAPK3	Mean Z-standardized log-normalized expression of pathway member genes
EGF Pathway	MAPK1	Mean Z-standardized log-normalized expression of pathway member genes
EGF Pathway	AKT1	Mean Z-standardized log-normalized expression of pathway member genes
EGF Pathway	PIK3CA	Mean Z-standardized log-normalized expression of pathway member genes
EGF Pathway	MTOR	Mean Z-standardized log-normalized

		expression of pathway member genes
PDGF Pathway	PDGFRA	Mean Z-standardized log-normalized expression of pathway member genes
PDGF Pathway	PDGFRB	Mean Z-standardized log-normalized expression of pathway member genes
PDGF Pathway	PDGFA	Mean Z-standardized log-normalized expression of pathway member genes
PDGF Pathway	PDGFB	Mean Z-standardized log-normalized expression of pathway member genes
PDGF Pathway	PIK3CA	Mean Z-standardized log-normalized expression of pathway member genes
PDGF Pathway	PIK3CB	Mean Z-standardized log-normalized expression of pathway member genes
PDGF Pathway	AKT1	Mean Z-standardized log-normalized expression of pathway member genes
PDGF Pathway	AKT2	Mean Z-standardized log-normalized expression of pathway member genes
PDGF Pathway	MAPK1	Mean Z-standardized log-normalized expression of pathway member genes
PDGF Pathway	MAPK3	Mean Z-standardized log-normalized expression of pathway member genes
PDGF Pathway	JAK1	Mean Z-standardized log-normalized expression of pathway member genes
PDGF Pathway	STAT3	Mean Z-standardized log-normalized expression of pathway member genes
FGF Pathway	FGFR1	Mean Z-standardized log-normalized expression of pathway member genes
FGF Pathway	FGFR2	Mean Z-standardized log-normalized expression of pathway member genes
FGF Pathway	FGFR3	Mean Z-standardized log-normalized expression of pathway member genes
FGF Pathway	FGF1	Mean Z-standardized log-normalized expression of pathway member genes
FGF Pathway	FGF2	Mean Z-standardized log-normalized expression of pathway member genes
FGF Pathway	FGF7	Mean Z-standardized log-normalized expression of pathway member genes
FGF Pathway	SPRY1	Mean Z-standardized log-normalized expression of pathway member genes
FGF Pathway	SPRY2	Mean Z-standardized log-normalized expression of pathway member genes
FGF Pathway	CRKL	Mean Z-standardized log-normalized expression of pathway member genes
FGF Pathway	GRB2	Mean Z-standardized log-normalized expression of pathway member genes
FGF Pathway	SOS1	Mean Z-standardized log-normalized expression of pathway member genes
FGF Pathway	MAPK3	Mean Z-standardized log-normalized expression of pathway member genes
Migration Score (keratinocyte)	MMP1	Mean Z-standardized log-normalized expression of pathway member genes
Migration Score (keratinocyte)	MMP3	Mean Z-standardized log-normalized expression of pathway member genes
Migration Score (keratinocyte)	MMP10	Mean Z-standardized log-normalized expression of pathway member genes
Migration Score (keratinocyte)	LAMC2	Mean Z-standardized log-normalized expression of pathway member genes
Migration Score (keratinocyte)	LAMB3	Mean Z-standardized log-normalized expression of pathway member genes
Migration Score (keratinocyte)	ITGA6	Mean Z-standardized log-normalized expression of pathway member genes
Migration Score (keratinocyte)	ITGB4	Mean Z-standardized log-normalized expression of pathway member genes

Migration Score (keratinocyte)	CDH1	Mean Z-standardized log-normalized expression of pathway member genes
Migration Score (keratinocyte)	VIM	Mean Z-standardized log-normalized expression of pathway member genes
Migration Score (keratinocyte)	FN1	Mean Z-standardized log-normalized expression of pathway member genes
Migration Score (keratinocyte)	TGFB1	Mean Z-standardized log-normalized expression of pathway member genes
Migration Score (keratinocyte)	RAC1	Mean Z-standardized log-normalized expression of pathway member genes
Migration Score (keratinocyte)	CDC42	Mean Z-standardized log-normalized expression of pathway member genes
